# Transcriptomic profiling of senescence effects on blood–brain barrier-related gene expression in brain capillary endothelial cells in a mouse model of paclitaxel-induced chemobrain

**DOI:** 10.1007/s11357-025-01561-5

**Published:** 2025-02-20

**Authors:** Roland Patai, Tamas Kiss, Rafal Gulej, Adam Nyul-Toth, Boglarka Csik, Siva Sai Chandragiri, Santny Shanmugarama, Stefano Tarantini, Anna Ungvari, Pal Pacher, Peter Mukli, Andriy Yabluchanskiy, Anna Csiszar, Zoltan Ungvari

**Affiliations:** 1 Vascular Cognitive Impairment, Neurodegeneration and Healthy Brain Aging Program, Department of Neurosurgery, University of Oklahoma Health Sciences Center, Oklahoma City, OK USA; 2 Oklahoma Center for Geroscience and Healthy Brain Aging, University of Oklahoma Health Sciences Center, Oklahoma City, OK USA; 3 Institute of Preventive Medicine and Public Health, Semmelweis University, Budapest, Hungary; 4 Pediatric Center, Semmelweis University, Budapest, Hungary; 5 HUN-REN-SU Cerebrovascular and Neurocognitive Diseases Research Group, 1094 Budapest, Hungary; 6 International Training Program in Geroscience, Doctoral College/Institute of Preventive Medicine and Public Health, Semmelweis University, Budapest, Hungary; 7 Department of Health Promotion Sciences, College of Public Health, University of Oklahoma Health Sciences Center, Oklahoma City, OK USA; 8 The Peggy and Charles Stephenson Cancer Center, University of Oklahoma Health Sciences Center, Oklahoma City, OK USA; 9 Laboratory of Cardiovascular Physiology and Tissue Injury, National Institute on Alcohol Abuse and Alcoholism, National Institutes of Health, Bethesda, MD USA

**Keywords:** Senescence, Chemotherapy, Chemotherapy-induced cognitive impairment, Aging, Vascular cognitive impairment, Aging, Dementia, Taxol, Cerebromicrovascular, VCID, Vascular cognitive impairment

## Abstract

Chemotherapy-induced cognitive impairment (CICI), commonly referred to as “chemobrain,” is a frequent and debilitating side effect experienced by cancer survivors treated with paclitaxel (PTX). Preclinical models have shown that PTX promotes cerebromicrovascular endothelial cell senescence, leading to chronic blood–brain barrier (BBB) disruption and neuroinflammation. Conversely, the elimination of senescent cells through senolytic therapies has been shown to restore BBB integrity, reduce neuroinflammation, and alleviate PTX-induced cognitive impairment. In this study, we tested the hypothesis that PTX-induced endothelial senescence alters gene expression patterns associated with BBB integrity. To investigate this, we analyzed a scRNA-seq dataset from the brains of mice treated with a clinically relevant PTX regimen alongside vehicle-treated control mice. We identified capillary endothelial cells by their distinct transcriptomic profiles and matched these profiles to known transcriptomic markers of cellular senescence. Our analysis confirmed that PTX induces senescence in capillary endothelial cells and revealed significant transcriptional alterations linked to impaired BBB function. In senescent endothelial cells, gene set enrichment analysis (GSEA) highlighted downregulated pathways associated with cell junction assembly and upregulated pathways involved in extracellular matrix remodeling and inflammatory signaling, including Vitronectin (VTN) and Pleiotrophin (PTN) pathways. Additionally, cell–cell communication analysis revealed reduced Junctional Adhesion Molecule (JAM) signaling, further implicating senescence in BBB disruption. These findings highlight endothelial senescence as a driver of BBB dysfunction through transcriptional changes and altered intercellular signaling. The enrichment of VTN and PTN pathways in the senescent state indicates a shift toward vascular remodeling and inflammation, exacerbating microvascular fragility and BBB disruption. Supported by prior experimental findings, this study suggests that targeting endothelial senescence and its downstream effects could mitigate PTX-induced BBB dysfunction and associated cognitive impairments. These results advance our understanding of CICI pathogenesis and provide a foundation for developing therapeutic strategies aimed at preserving vascular integrity.

## Introduction

Chemotherapy-induced cognitive impairment (CICI), often termed “chemobrain,” is a frequent and challenging side effect in cancer survivors treated with chemotherapeutic agents [1–3] such as paclitaxel (PTX). Approximately 30–50% of patients experience cognitive dysfunction, with deficits in memory, attention, and executive function, which can persist long after treatment and significantly impair quality of life and treatment outcomes [1, 3]. Despite advances in cancer care, strategies to mitigate or prevent CICI remain limited, highlighting the need for a better understanding of the underlying mechanisms to develop effective interventions.

Recent preclinical studies have demonstrated that PTX significantly impacts brain microvascular endothelial cells by promoting cellular senescence, which leads to complex microvascular dysfunction [4]. Initial clinical studies support these findings, showing that patients treated with PTX exhibit compromised cerebral blood flow (CBF) regulation [5]. In preclinical models, PTX-induced endothelial senescence in the cerebromicrovascular system is associated with several detrimental effects, including disruption of the blood–brain barrier (BBB) [4]. The pathophysiological consequences of endothelial senescence and BBB disruption are profound, as cerebral microvascular endothelial cells are essential to the BBB, where they form extensive tight junctions that prevent leakage of blood-borne factors into the brain parenchyma. While temporary BBB opening with adjuvant treatment has been explored to enhance chemotherapy efficacy for treating brain metastases or brain tumors [6–8], maintaining BBB integrity remains essential for preserving normal brain function [9–11]. The BBB supports structural and functional brain connectivity, synaptic activity, and cognitive processing, all of which are crucial for healthy neural function [9, 10]. Disruption of this barrier can lead to unwanted neuroinflammation, synaptic dysfunction, and neurodegeneration, ultimately impairing cognitive health [9, 10]. Accordingly, PTX-induced BBB disruption in mouse models has been directly linked to increased neuroinflammation, which, along with microvascular rarefaction, endothelial dysfunction, and impaired neurovascular coupling, collectively contributes to cognitive decline resembling CICI symptoms observed in cancer survivors [4]. Importantly, studies have shown that selectively eliminating senescent cells through senolytic therapies can partially restore BBB integrity, reduce neuroinflammation, and improve cognitive function in PTX-treated models [4]. Furthermore, data from irradiated mice [12] and BubR1 hypomorphic (BubR1^(H/H)^) mice [13], which exhibit senescence-dependent phenotypes, further supports the causal role of cellular senescence in BBB disruption. These findings underscore the role of senescent endothelial cells in mediating CICI and suggest that targeting these cells could offer a promising therapeutic approach for mitigating PTX-induced neurovascular and cognitive impairments [4].

Despite these advances, a critical gap in knowledge remains. It is unclear whether PTX-induced endothelial senescence directly disrupts BBB integrity by altering the transcriptomic expression of BBB-related genes. Such transcriptional changes could, in theory, contribute to the increased permeability observed in BBB disruption. Alternatively, BBB compromise may arise from senescence-associated secretory phenotype (SASP) factors [11], phosphorylation changes affecting junctional proteins, or other post-transcriptional mechanisms. Understanding whether gene expression changes in senescent endothelial cells are directly associated with BBB integrity could clarify the pathways through which PTX-induced senescence contributes to CICI and guide therapeutic approaches targeting specific mechanisms.

This study aims to investigate whether PTX-induced endothelial senescence in a mouse model of chemobrain is associated with transcriptomic changes in BBB-related gene expression. Using single-cell RNA sequencing (scRNA-seq) data from the brains of PTX-treated and control mice, we analyzed capillary endothelial cells for transcriptomic signatures indicative of senescence. We hypothesized that senescent endothelial cells would exhibit altered expression of genes crucial for BBB integrity, including those involved in cell junction assembly.

## Materials and methods

### Study design and data source

This study utilized a dataset derived from a previously reported cohort in which the effects of PTX on BBB integrity and endothelial cell senescence were investigated in a murine model of CICI [4]. The dataset includes scRNA-seq data from brains isolated from PTX-treated and vehicle-treated control mice, which allowed for a detailed transcriptomic analysis of capillary endothelial cells and their senescent profiles. In the following sections, we provide a brief description of the experimental animals, treatment protocols, and scRNA-seq methodologies used in this study [4].

### Experimental animals and PTX treatment protocol

In our study, we used a novel transgenic mouse model (p16-3MR mice [14]) engineered to carry a fusion protein (3MR) under the control of the p16^*Ink4a*^ promoter enabling the identification of senescent cells as we reported [4]. Three-month-old male p16-3MR mice received paclitaxel (PTX) at a dose of 5 mg/kg/day intraperitoneally or vehicle (DMSO + saline) in two cycles, with each cycle consisting of five consecutive days of treatment followed by a 7-day interval between cycles. Six months following the PTX protocol, the mice underwent assessments of cognitive function (radial arm water maze testing) and BBB integrity (two photon microscopy-based intravital imaging) [4] before being euthanized for tissue collection. The 3MR fusion protein includes a monomeric red fluorescent protein (mRFP), which allowed for the quantitative assessment of senescence burden among various brain cell populations using flow cytometry. These methods enabled us to demonstrate that PTX significantly increases endothelial senescence, which is associated with BBB disruption and a marked decline in cognitive performance [4]. All animal procedures adhered to ethical standards and were approved by the Institutional Animal Care and Use Committee of the University of Oklahoma Health Sciences Center (OUHSC).

### Single-cell RNA sequencing (scRNA-seq) and transcriptomic analyses

The sequencing procedures were performed in the referenced study [4], and here we provide a brief overview of the methodology along with the transcriptomic analyses conducted in this study. To assess PTX-induced transcriptomic changes in capillary endothelial cells, scRNA-seq was performed on brain tissues harvested from PTX-treated and control mice 6 months post-treatment.

#### Tissue processing and cell isolation

Brains from PTX-treated mice (*n* = 5) were rapidly removed upon cardiac perfusion with ice-cold PBS, rinsed in ice-cold PBS, and minced into approximately 1 mm3 pieces [4]. Single-cell suspensions were prepared from the brain samples using a modified version of the protocol previously established for Fluorescence-Activated Cell Sorting (FACS) studies. Briefly, brain samples were enzymatically digested, mechanically disintegrated, depleted of myelin, and cleared using Debris Removal Solution (Miltenyi Biotech) to eliminate extracellular material and cellular debris. The resulting cell pellets were then stained with SYTOX™ Green Nucleic Acid Stain (Invitrogen) to label dead cells. To enrich the sample with viable cells, dead cells were removed through FACS using the low-pressure WOLF Cell Sorter™ (NanoCellect), which allowed for the collection of a high-quality, intact cell suspension ideal for transcriptomic studies. Cells were maintained on ice until sequencing, ensuring cell integrity and suitability for single-cell RNA sequencing analysis. This method optimizes the quality of isolated cells, enhancing the reliability of subsequent transcriptomic analysis.

#### Single-cell RNA sequencing (scRNA-seq)

All samples were processed simultaneously through each step to ensure consistency in generating stable cDNA libraries. After tissue dissociation, cells were diluted in ice-cold PBS containing 0.4% BSA to maintain cell viability. The cells were then loaded onto a Chromium Single Cell 3′ Chip (10 × Genomics, Pleasanton, California) and processed according to the manufacturer’s instructions. Library construction was carried out using the Chromium Single Cell 3′ Library & Gel Bead Kit v2 (Catalog# 120267, 10 × Genomics, Pleasanton, California). The libraries were pooled based on their molar concentrations and sequenced on one high-output lane of the NovaSeq 6000 instrument (Illumina, San Diego, California). For data processing, we used the 10 × Genomics Cell Ranger (v3.0.2) pipeline to demultiplex samples, process barcodes, align and filter reads, and generate feature-barcode matrices according to the manufacturer’s instructions. Reads were mapped to the mm10 mouse transcriptome reference (v.1.2.0) provided by 10 × Genomics.

#### Preprocessing of single cell data

Downstream analyses of the Cell Ranger output were conducted using the Seurat (v5.1) workflow [4, 15], implemented as an R package (R, v4.4.1) [16, 17]. All samples were merged into a single Seurat object for subsequent bioinformatic analysis. Our raw dataset consisted of 27,524 cells.

First, low-quality cells were removed using thresholds set by individual samples. The following parameters were used: the number of unique molecules detected (top and bottom 10 percentile was removed), the number of unique genes detected (top and bottom 10 percentile was removed) and the percentage of reads mapping to the mitochondrial genome (top 20 percentile was removed) in each cell.

Normalization was performed using the SCTransform algorithm [18] implemented natively in the Seurat workflow [4, 19]. The variable “percentage of reads mapping to the mitochondrial genome” was regressed out. To speed up our calculation, we used the glmGamPoi method [20] during this step.

Principal component analysis (PCA) was performed on the scaled data, and the top 20 principal components (PCs) were used for further analysis, including clustering and visualization. The clustering was performed using the unbiased Louvain algorithm implemented in the Seurat workflow, using a resolution parameter of 0.07. Clusters representing fewer than 3% of all cells were excluded from downstream analysis. Cell clusters were annotated by inspecting the expression of previously validated canonical cell type markers [4, 21].

Finally, to visualize the cell clusters, a non-linear dimensionality reduction technique called Uniform Manifold Approximation and Projection (UMAP) was employed using the uwot method (v0.2.2) called by the Seurat workflow.

#### Identification of senescent endothelial cells

For the downstream analysis, endothelial cells identified by unsupervised clustering and canonical marker genes (Tables 1 and 2) were subset and saved to a separated Seurat object. Before the automated cell type annotation was performed using the SingleR Bioconductor package [22] and a publicly available reference single-cell dataset [23], the principal component analysis was recomputed on this subset. To validate the result of the automatic cell type annotations, canonical endothelial markers were also inspected manually.

**Table 1:** List of cell-type specific markers [40]

Cell type	Symbol	ENTREZ ID	Gene name
Endothelial cells	*Cldn5*	12741	claudin 5
Endothelial cells	*Flt1*	14254	FMS-like tyrosine kinase 1
Endothelial cells	*Slco1a4*	28250	solute carrier organic anion transporter family, member 1a4
Microglia cells	*Cx3cr1*	13051	chemokine (C-X3-C motif) receptor 1
Microglia cells	*P2ry12*	70839	purinergic receptor P2Y, G-protein coupled 12
Microglia cells	*Tmem119*	231633	transmembrane protein 119
Pericytes	*Pdgfrb*	18596	platelet derived growth factor receptor, beta polypeptide
Pericytes	*Kcnj8*	16523	potassium inwardly-rectifying channel, subfamily J, member 8
Oligodendrocytes	*Cldn11*	18417	claudin 11
Oligodendrocytes	*Cnp*	12799	2′,3′-cyclic nucleotide 3′ phosphodiesterase
Smooth muscle cells	*Acta2*	11475	actin, alpha 2, smooth muscle, aorta
Smooth muscle cells	*Myh11*	17880	myosin, heavy polypeptide 11, smooth muscle
Astrocytes	*Aqp4*	11829	aquaporin 4
Astrocytes	*Slc1a2*	20511	solute carrier family 1 (glial high affinity glutamate transporter), member 2

**Table 2:** List of endothelial cell (EC) sub-cluster specific genes [23]

Endothelial cell sub-clusters	Gene symbol	ENTREZ ID	Gene name
Arterial/arteriolar EC	*Gkn3*	68888	gastrokine 3
Arterial/arteriolar EC	*Stmn2*	20257	stathmin-like 2
Arterial/arteriolar EC	*Clu*	12759	clusterin
Venous EC	*Icam1*	15894	intercellular adhesion molecule 1
Venous EC	*Slc38a5*	209837	solute carrier family 38, member 5
Venous EC	*Tmsb10*	19240	thymosin, beta 10
Capillary EC	*Cxcl12*	20315	chemokine (C-X-C motif) ligand 12
Capillary EC	*Rgcc*	66214	regulator of cell cycle
Capillary EC	*Spock2*	94214	sparc/osteonectin, cwcv and kazal-like domains proteoglycan 2

Senescent endothelial cells were identified using the previously published SenMayo gene set [24] alongside Cdkn2a, a canonical biomarker of cellular senescence. AUCell enrichment scores [25] for the SenMayo gene set [24] were calculated using the escape Bioconductor package [26]. Cells expressing Cdkn2a or displaying an AUCell enrichment score greater than 0.155 were classified as senescent.

Mouse specific SenMayo gene set from the Molecular Signatures Database (MSigDB) accessed via Bioconductor Annotation Hub was used [27].

#### Cell-to-cell interaction analysis

To investigate intercellular communication networks within the single-cell transcriptomic data, we utilized the CellChat package (v2.1.2) [28]. CellChat infers signaling pathways by identifying known ligand-receptor interactions and modeling communication probabilities using a probabilistic framework. The built-in mouse CellChatDB v2 was used as a reference.

Among the PTX-treated cells, an intercellular communication analysis was performed, separately for non-senescent and senescent endothelial cells, analyzing their interactions with the five other cell types present in the dataset. To identify the signaling changes between these two inferred interaction networks, we used the netAnalysis_signalingChanges_scatter function with default parameters, highlighting differences in signaling pathways associated with senescence.

#### Gene set enrichment analysis

To assess the impact of PTX-induced senescence on BBB-related gene expression, gene set enrichment analysis (GSEA) was performed using gene sets linked to BBB function selected from the Molecular Signatures Database (MSigDB) accessed via Bioconductor Annotation Hub [27]. To calculate single cell-level enrichment scores, AUCell algorithm was used, implemented in the *escape* Bioconductor package [26]. To compare senescent and non-senesces endothelial cells among the paclitaxel-treated cells, the Wilcoxon Rank Sum was used. All visualizations for GSEA results and other analyses were generated using ggplot2.

## Results

### PTX induces senescence in capillary endothelial cells

**Fig. 1:**
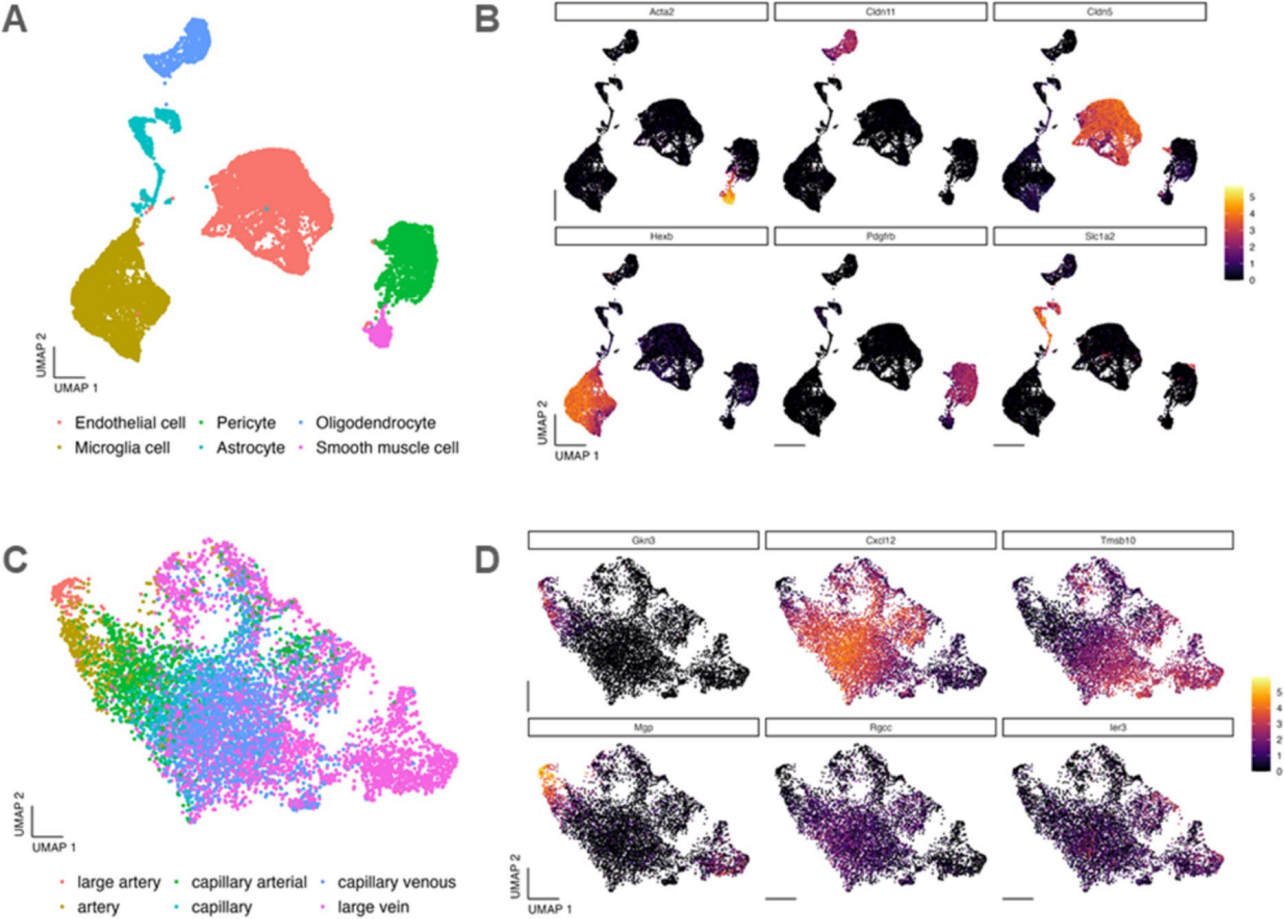
Identification of capillary endothelial cells in the brains of PTX-treated mice**. A** Identification of brain endothelial cells using differentially expressed marker genes obtained from scRNA-seq data. Shown is a two-dimensional UMAP plot with clusters colored by cellular identity. Cluster identities were assigned based on previously validated marker genes. **B** Expression profiles of selected marker genes across the identified clusters, illustrating the specificity of marker expression and confirming the transcriptional identity of each cell type. **C** Classification of capillary, venous, and arterial endothelial cells based on a reference dataset. The UMAP plot highlights endothelial subclusters, with each phenotype color-coded for clear differentiation. The marker genes used to define each subcluster were reported in previous studies [4]. **D** Visualization of the expression levels of selected endothelial subtype-specific marker genes within endothelial cells, demonstrating the phenotypic diversity across endothelial subtypes and providing a detailed view of transcriptional specialization

After the quality control steps, the final dataset comprised 16,396 cells. As we expected, with the combination of the unsupervised Louvain clustering and the inspection of the established, previously validated cell type markers (Table 1), we were able to identify endothelial cells (*n* = 7492), microglia (*n* = 4274), pericytes (*n* = 2371), astrocytes (*n* = 924), oligodendrocytes (*n* = 907), and smooth muscle cells (*n* = 428) in our dataset (Fig. 1A, B). Figure 1B illustrates the expression patterns of the most important canonical marker genes within the clusters, confirming the distinct transcriptional identities of the identified cell types.

For the further downstream analysis, we focused solely on the endothelial cells. To distinguish capillary endothelial cells from arterial and venous endothelial cells (Table 2), we performed a reference-based automated annotation with SingleR package v2.6.0 (Fig. 1C). The results of this reference-based cluster annotations were validated by examining the expression of canonical endothelial markers, which confirmed the accuracy of the cluster identities [4, 23] (Fig. 1C, D).

**Fig. 2:**
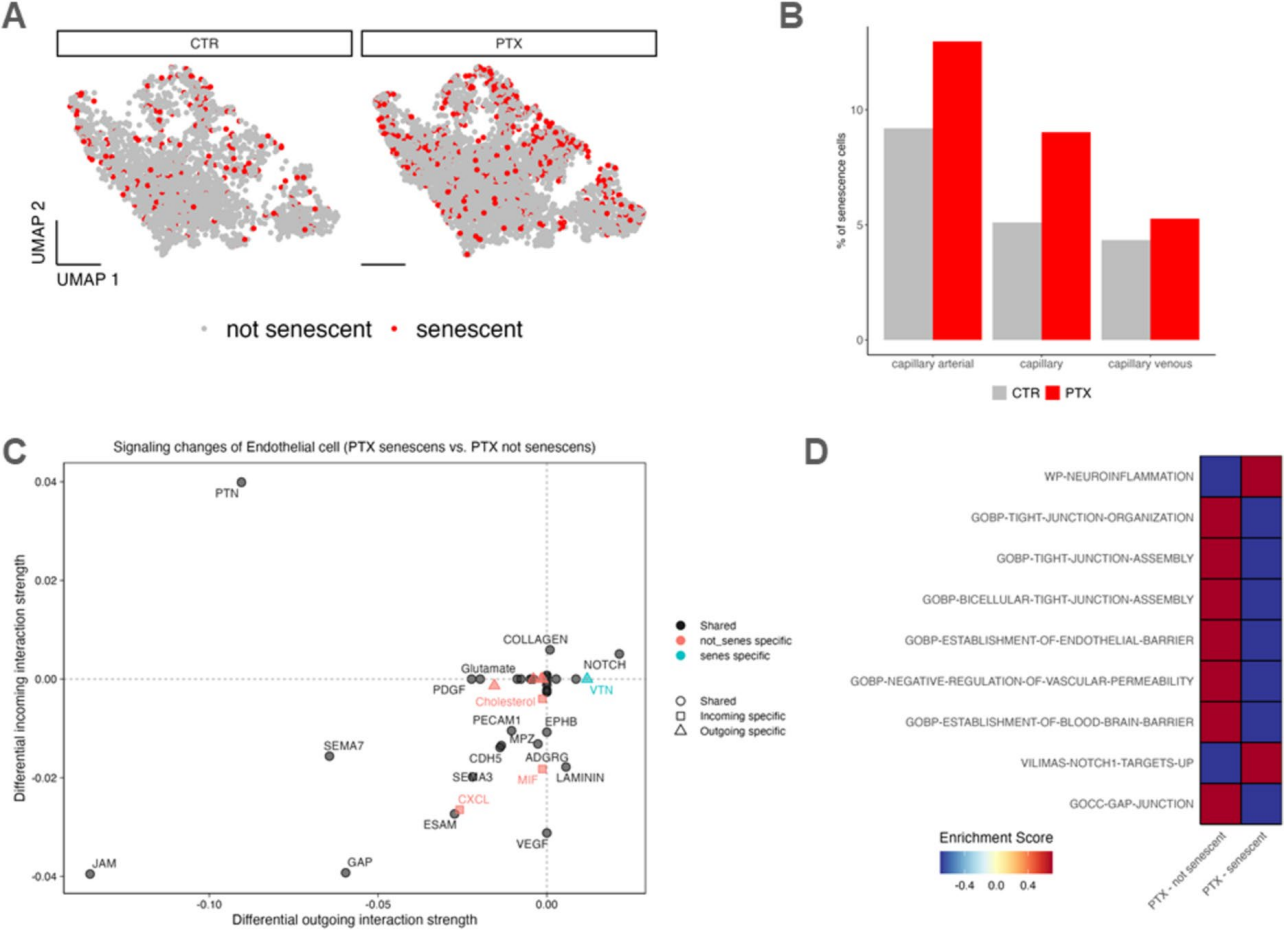
Characterization of senescence-related phenotypic shifts in cerebromicrovascular endothelial cells**. A** UMAP plots showing endothelial cells with high expression of senescence markers in the brains of PTX-treated and control mice. Senescent endothelial cells were identified using the previously published SenMayo gene set alongside Cdkn2a. **B** Bar charts illustrating the percentage of senescent arteriolar, capillary, and venular endothelial cells in the brains of PTX-treated and control mice. The analysis highlights an increased prevalence of senescence among endothelial cell subtypes in PTX-treated mice compared to controls. **C** Scatter plot depicting senescence-related signaling changes in endothelial cells. The differential interaction strengths of senescent versus non-senescent endothelial cells are shown, with the x-axis representing changes in outgoing interaction strength and the y-axis indicating changes in incoming interaction strength. Key cellular processes influenced by senescence, such as gap junctional communication (GAP) and junctional adhesion molecules (JAM), are labeled to emphasize significant shifts in endothelial communication dynamics driven by PTX-induced senescence. **D** Heatmap of gene set enrichment analysis (GSEA) results for capillary endothelial cells comparing senescent and non-senescent states. Enrichment scores for biological pathways related to endothelial barrier function are shown, with red indicating positive enrichment and blue indicating negative enrichment. This visualization highlights the transcriptional changes in senescent endothelial cells associated with PTX treatment, underscoring the molecular pathways impacted by cellular senescence

To identify senescence within the endothelial cell population, we analyzed the expression of Cdkn2a, a canonical senescence biomarker, alongside the SenMayo gene set of senescence-associated markers [24]. Using the Cdkn2a marker and the gene set enrichment analysis, we identified senescent endothelial cells in the brains of PTX-treated mice (Fig. 2A). These senescent capillary endothelial cells were notably enriched in the PTX-treated group, demonstrating a significant accumulation induced by PTX treatment (Fig. 2B). These findings highlight the impact of PTX on promoting senescence within the capillary endothelial cell population [4].

### Signaling changes in capillary endothelial cells following PTX treatment: comparison of senescent and non-senescent states

To compare cell–cell communication dynamics of senescent and non-senescent endothelial cells in PTX-treated brains, we utilized CellChat, a computational tool designed to infer and quantify cell–cell communication networks from single-cell RNA sequencing data. This analysis enabled a detailed assessment of signaling interactions within the brain’s microvascular environment. Both incoming and outgoing interaction strengths of endothelial cells were evaluated. The change of interaction scores between PTX-induced senescent and non-senescent endothelial cells was calculated and visualized in a two-dimensional scatter plot (Fig. 2C). Our analysis revealed a distinct reorganization of cell–cell communication networks in senescent capillary endothelial cells compared to their non-senescent counterparts.

A notable finding was the significant reduction in Junctional Adhesion Molecule (JAM) signaling. This loss of interaction strength was observed in endothelial-endothelial communication and between endothelial cells and pericytes, microglia, and astrocytes. The disruption of JAM signaling suggests impaired cell–cell adhesion and compromised BBB integrity. Similarly, a decrease in Gap Junction (GAP) communication highlights impaired intercellular coordination among endothelial cells and between endothelial and supporting cell types.

Additional pathways with diminished signaling in senescent cells included Semaphorin-7A (SEMA7), which plays a role in cytoskeletal remodeling, and Pleiotrophin (PTN), involved in matrix remodeling and angiogenesis. These reductions may reflect the broader loss of endothelial functionality in the senescent state.

Conversely, certain pathways were enriched in the senescent state. Vitronectin (VTN), an extracellular matrix-related pathway, was specifically upregulated in senescent endothelial cells, indicating enhanced matrix remodeling activity. These changes align with a senescence-associated phenotype characterized by increased extracellular matrix turnover and pro-remodeling signals. Notably, Cyclophilin A (CypA) emerged as a senescence-specific pathway with increased signaling in senescent endothelial cells. This pathway, often linked to inflammatory responses, suggests a shift toward a pro-inflammatory environment in senescent cells.

These findings highlight a complex rewiring of signaling pathways in PTX-induced senescent endothelial cells. Reduced JAM and GAP signaling indicate impaired vascular homeostasis and barrier integrity, while increased PTN and VTN signaling point to a remodeling-dominant phenotype. The sustained presence of inflammatory and matrix remodeling signals, exemplified by CypA and Vitronectin, underscores the multifactorial nature of endothelial dysfunction in senescence. This analysis supports the hypothesis that PTX-induced senescence disrupts vascular stability by tipping the balance between pro-repair and pro-inflammatory signals. Such changes contribute to endothelial dysfunction, microvascular fragility, and BBB disruption, which are hallmarks of CICI.

### Pathway enrichment analysis in PTX-treated senescent and non-senescent capillary endothelial cells

To assess functional changes in capillary endothelial cells associated with PTX-induced senescence, we performed pathway enrichment analysis on our single-cell RNA sequencing data. Gene sets available in the MSiDB (Table 3) were compared between PTX-treated senescent and non-senescent endothelial cells using the AUCell algorithm. The results are displayed as a heatmap, where the enrichment score indicates the relative activity of specific pathways in each cell state (Fig. 2D). This analysis revealed substantial differences in key pathways between senescent and non-senescent capillary endothelial cells. We found enhanced neuroinflammatory signaling in senescent cells, indicating a pro-inflammatory senescence-associated phenotype that may contribute to vascular and cognitive dysfunction. Upregulation of Notch1 signaling targets in senescent endothelial cells suggests an altered angiogenic and repair response in senescent cells, reflecting their maladaptive interaction with the vascular environment. Notch signaling has also been implicated in the mediation of secondary senescence [29]. Significant downregulation in tight junction-related pathways indicates compromised endothelial barrier integrity and may explain BBB disruption observed in our recent studies [4]. Reduced activity in pathways supporting blood–brain barrier integrity suggests senescence-driven vascular dysfunction in the brain. Downregulation of gap junction communication highlights impaired intercellular signaling in senescent cells, further disrupting vascular homeostasis. This analysis underscores the dual impact of PTX-induced senescence: a pro-inflammatory shift alongside a significant loss of endothelial barrier function. The enrichment of neuroinflammation and Notch1 signaling in senescent cells suggests maladaptive vascular responses that exacerbate dysfunction. Conversely, the downregulation of tight junction assembly, gap junction signaling, and blood–brain barrier pathways highlights the structural and functional deterioration of endothelial integrity. These findings provide mechanistic insights into PTX-induced vascular aging and suggest potential therapeutic targets for mitigating endothelial senescence.

**Table 3:** MSigDB datasets used in the analysis

Standard name	Collection
SAUL_SEN_MAYO	Curated (M2)
WP-NEUROINFLAMMATION	Curated (C2)
GOBP-TIGHT-JUNCTION-ORGANIZATION	Gene Ontology (M5)
GOBP-TIGHT-JUNCTION-ASSEMBLY	Gene Ontology (M5)
GOBP-BICELLULAR-TIGHT-JUNCTION-ASSEMBLY	Gene Ontology (M5)
GOBP-ESTABLISHMENT-OF-ENDOTHELIAL-BARRIER	Gene Ontology (M5)
GOBP-NEGATIVE-REGULATION-OF-VASCULAR-PERMEABILITY	Gene Ontology (M5)
GOBP-ESTABLISHMENT-OF-BLOOD–BRAIN-BARRIER	Gene Ontology (M5)
VILIMAS-NOTCH1-TARGETS-UP	Curated (C2)
GOCC-GAP-JUNCTION	Gene Ontology (M5)

## Discussion

This study provides new insights into the mechanisms by which PTX-induced cerebromicrovascular endothelial senescence may affect BBB integrity in the context of CICI. Using scRNA-seq in a mouse chemobrain model, we demonstrated that PTX induces cellular senescence in cerebromicrovascular endothelial cells, including capillary endothelial cells [4]. Importantly, we identified significant transcriptomic changes in senescent capillary endothelial cells that correlate with impaired BBB function, including alterations in pathways critical for tight junction integrity, transporter function, and inflammatory signaling.

Our data revealed the downregulation of tight junction-related genes and pathways in senescent endothelial cells, suggesting a direct transcriptional basis for the compromised barrier function. Our analysis of cell–cell communication dynamics further highlighted the profound rewiring of signaling networks in senescent endothelial cells. Notably, we observed a significant reduction in signaling strength for JAM, a pathway critical for maintaining BBB integrity. The diminished interaction strength in JAM signaling between endothelial cells and other cell types aligns with the observed disruption in tight junction integrity and increased BBB permeability [4]. Conversely, pathways like PTN and VTN were enriched in senescent endothelial cells, indicating a shift toward extracellular matrix remodeling and pro-inflammatory signaling. PTN exhibited sustained outgoing signaling toward supporting cells such as smooth muscle cells, suggesting its role in stress-induced vascular remodeling. VTN’s enrichment highlights its contribution to senescence-associated extracellular matrix turnover and adhesion changes, processes that likely exacerbate microvascular fragility and barrier dysfunction in the context of PTX treatment. Extracellular matrix components like collagen and laminin were consistently engaged in both senescent and non-senescent states, albeit with differing roles. In senescent cells, these pathways may reflect a maladaptive response aimed at compensating for structural deficits caused by vascular stress. These findings add a new dimension to our understanding of how PTX-induced senescence exacerbates cognitive impairment by disrupting microvascular homeostasis. The interplay between reduced JAM and GAP signaling and enhanced PTN and VTN signaling underscores a complex senescence-driven remodeling phenotype that destabilizes the BBB.

Our findings build on previous studies showing that PTX-induced endothelial senescence contributes to a multifaceted pattern of microvascular dysfunction, including impaired neurovascular coupling (NVC), microvascular rarefaction, and BBB disruption associated with increased neuroinflammation [4]. These factors collectively exacerbate cognitive decline by disrupting the metabolic support and homeostatic protection that the cerebral microcirculation provides to neurons [9, 10, 30–36]. The brain’s microvascular network is essential for delivering oxygen, glucose, and other nutrients to neurons while removing waste products and maintaining a stable environment critical for neuronal function. When this network is compromised, as seen with chemotherapy-induced endothelial senescence, the resulting reduction in metabolic support and increased BBB permeability lead to neuroinflammation, oxidative stress, and impaired synaptic function [4, 11, 37, 38]. These alterations collectively exacerbate neurodegenerative processes, ultimately contributing to the cognitive deficits observed in conditions like CICI.

This study aligns with evidence suggesting that cellular senescence, particularly in capillary endothelial cells, is a key driver of microvascular dysfunction that predisposes the brain to neurodegeneration and cognitive impairment [12, 13, 35, 39–43]. Notably, depletion of senescent cells in preclinical models has been shown to restore BBB integrity, capillarization, and NVC responses, ultimately improving cognitive outcomes [4, 12, 35, 44]. These findings emphasize the therapeutic potential of targeting senescence in CICI.

While our results underscore the role of senescence-related transcriptional changes in BBB disruption, it is essential to consider complementary mechanisms. Senescent cells are known to release a host of pro-inflammatory factors, matrix-degrading enzymes, and other bioactive molecules collectively termed the senescence-associated secretory phenotype (SASP) [41, 45–50]. SASP factors, such as cytokines IL-6 and IL-1β, can indirectly increase BBB permeability by creating a pro-inflammatory environment that exacerbates vascular dysfunction and neuroinflammation. Such factors may interact with neighboring endothelial cells or astrocytes, promoting a cascade of signaling events that compromise BBB integrity. Additionally, disruption of tight and adherens junctions in the BBB may occur due to post-translational modifications, such as phosphorylation, of junctional proteins [51–55]. Activation of kinases such as Src, PKC, and MAPKs can phosphorylate key proteins, including occludin, claudins, and VE-cadherin, leading to junctional disassembly and increased permeability [51–55]. These modifications provide a complementary mechanism through which PTX-induced endothelial senescence may disrupt the BBB in addition to transcriptional changes.

In addition, the endothelial syncytium enables senescent endothelial cells to influence nearby cells through paracrine signaling or direct communication via gap junctions. This intercellular communication may further amplify the effects of senescence on microvascular integrity. Future investigations should explore these pathways to fully elucidate the multifactorial nature of BBB disruption in CICI.

To advance our understanding, further studies are warranted to validate these transcriptomic findings at the protein level, particularly through Western blotting or immunofluorescence staining for key BBB structural components such as occludin, claudins, and VE-cadherin and functional assays of BBB permeability. Investigations focused on SASP factor profiles, phosphorylation states of junctional proteins, and epigenetic changes could provide valuable insights into the post-transcriptional and non-cell-autonomous effects of senescence on the BBB. Therapeutic interventions targeting the identified pathways, such as anti-inflammatory treatments, could provide valuable insights into their efficacy in preserving BBB integrity and mitigating cognitive decline. While this study focused on endothelial cells due to their primary role in forming the BBB, other cells, including pericytes, astrocytes, and microglia, likely contribute to PTX-induced BBB dysfunction. Pericytes play a crucial role in maintaining endothelial barrier function, and their loss or dysfunction could exacerbate BBB leakage. Astrocytes regulate BBB homeostasis via endfeet interactions and release of vasoactive mediators, and microglia activation in response to PTX may amplify neuroinflammation and compromise BBB integrity. Exploring the roles of these types in PTX-induced BBB disruption may yield a more comprehensive understanding of the BBB’s response to chemotherapy.

Endothelial senescence is a hallmark of vascular aging and has been implicated in neurodegenerative disorders such as Alzheimer’s disease [56–58]. The endothelial dysfunction observed in PTX-treated mice shares similarities with age-related cerebrovascular changes, including BBB breakdown, increased neuroinflammation, and microvascular rarefaction (Ungvari and coworkers, submitted, 2024). This raises the intriguing possibility that PTX-treated mice could serve as a model for studying mechanisms of age-related BBB deterioration. Future studies should compare PTX-induced endothelial senescence to aging-associated endothelial dysfunction to determine shared and distinct molecular pathways driving BBB disruption.

In summary, this study highlights the critical role of endothelial cell senescence in PTX-induced BBB disruption and underscores its contribution to the pathogenesis of CICI. Our findings demonstrate that PTX-induced senescence in capillary endothelial cells is associated with significant transcriptomic changes that impair BBB integrity. The ability to selectively eliminate senescent cells through senolytic therapies offers a promising approach to preserving BBB integrity and cognitive function in the context of PTX treatment [4]. Both genetic and pharmacologic depletion of senescent cells have been shown to restore BBB integrity, reduce neuroinflammation, and prevent cognitive decline in preclinical models [4]. These results align with growing evidence that senolytics can mitigate the adverse effects of cellular senescence on microvascular function in the aging brain [35, 59]. In addition to direct senolytic strategies, targeting specific senescence-associated signaling pathways may offer therapeutic benefits. Pharmacological inhibitors of CypA have shown promise in reducing endothelial inflammation and could be tested in PTX-induced BBB dysfunction models [60, 61]. Similarly, strategies aimed at stabilizing tight junctions, such as Src kinase inhibitors to prevent occludin and claudin phosphorylation, may help maintain BBB integrity in the presence of endothelial senescence. Matrix remodeling inhibitors targeting vitronectin or pleiotrophin signaling could further reduce vascular instability. The translational potential of these approaches warrants investigation in preclinical models of CICI.

Importantly, chemotherapy can also cause senescence in lingering tumor cells and stromal cells and the potential effects of senolytics in that regard require careful consideration. Senolytic treatments could theoretically clear these senescent tumor cells, reducing the risk of recurrence. However, this approach comes with caveats. Chemotherapy-induced senescence in tumor cells may act as a barrier to proliferation. Removing these cells prematurely might undermine the benefits of senescence as a tumor-suppressive mechanism. Given these complexities, the integration of senolytics into post-chemotherapy care requires a nuanced approach [62, 63]. Further elucidation of the molecular pathways involved in BBB dysfunction will enhance the development of targeted therapies to address chemotherapy-induced cognitive impairment and its associated vascular dysfunctions.
